# Development of a Three Dimensional Neural Sensing Device by a Stacking Method

**DOI:** 10.3390/s100504238

**Published:** 2010-04-28

**Authors:** Chih-Wei Chang, Jin-Chern Chiou

**Affiliations:** 1 Department of Electrical Engineering, National Chiao Tung University, No. 1001, University Road, Hsinchu City 30010, Taiwan; E-Mail: cwchang.ece94g@nctu.edu.tw; 2 School of Medical, China Medical University, No.91, Hsueh-Shih Road, Taichung, Taiwan

**Keywords:** microassembly, microprobe array, three dimensional probe array

## Abstract

This study reports a new stacking method for assembling a 3-D microprobe array. To date, 3-D array structures have usually been assembled with vertical spacers, snap fasteners and a supporting platform. Such methods have achieved 3-D structures but suffer from complex assembly steps, vertical interconnection for 3-D signal transmission, low structure strength and large implantable opening. By applying the proposed stacking method, the previous techniques could be replaced by 2-D wire bonding. In this way, supporting platforms with slots and vertical spacers were no longer needed. Furthermore, ASIC chips can be substituted for the spacers in the stacked arrays to achieve system integration, design flexibility and volume usage efficiency. To avoid overflow of the adhesive fluid during assembly, an anti-overflow design which made use of capillary action force was applied in the stacking method as well. Moreover, presented stacking procedure consumes only 35 minutes in average for a 4 × 4 3-D microprobe array without requiring other specially made assembly tools. To summarize, the advantages of the proposed stacking method for 3-D array assembly include simplified assembly process, high structure strength, smaller opening area and integration ability with active circuits. This stacking assembly technique allows an alternative method to create 3-D structures from planar components.

## 1. Introduction

In recent years, advance micromachined/assembled micro probe arrays with electrical stimulation/recording ability have come to play an essential role in exploring central neural systems. Simultaneous observation of a larger number of cell activities has become the general requirement to understand the nervous system [1]. Advances in neuroscience and neuroprosthetics now require microelectrode arrays that are able to access numerous neurons simultaneously with high spatial resolution [2]. Recording of the extracellular action potentials has been accomplished by surgically implanting neural probes into the target neurons of interest, which resulted from neural activities [3]. Probes that could insert a large number of recording sites into neural tissues with minimal tissue damage are therefore needed. Also, the design of the probe arrays should be optimized for an experimental purpose that an electrode diameter of a few micrometers could support single-unit recording [4].

The traditional micro probes, which are made from insulated metal wires and glass micropipettes, cannot provide simultaneously multi-channel recording. The main reason is that the traditional devices function as only a single site on a single probe shaft. Some previous studies have improved the problem by thin-film lithography-based micromachining techniques since 1960s.

High-density probe arrays yielded insights into the organization and function of the neural system [5]. Silicon [6], glass [7], polymer [8] and sapphire [9] substrates have been employed as thin-film electrode probe planks. The thin-film silicon micro probe was developed many years ago for neuroscience and neural prostheses [10]. It has also been widely characterized electrically [11] and mechanically [12] for probe scaling [13], insertion force [14], tissue strain [15] and chronic brain responses [16]. The studies mentioned above provide detailed multi-channel recordings along a single plane, but lacked of full cell activity information in 3-D space [17].

To access the full cell activity that originates in the target tissue, three dimensional microprobe arrays are strongly required with precisely controlled dimensions and front-end circuitry compatibility. In other words, to achieve detailed studies of neural networks and implementation of neural prostheses, we need to access three-dimensional volumes of tissue with three-dimensional distributed recording sites. In modern neural system researches, 3D microprobe array allows the recording and mapping of the neural signal network and interconnections among the 3D brain structure. The recording and mapping would be impossible to achieve by using 2-D planar arrays [17].

Currents methods of fabricating three-dimensional microprobe array structures can be summarized as follows:

1. Silicon bulk etched microprobe array
2. polymer-constructed array
3. Creating 3-D arrays by the assembling of 2-D parts

For the silicon bulk etched out-of-plane microprobe arrays, every probe shaft in the array only functions as a single recording site [18]. The total number of the recording sites was limited when high recording density and number are strongly required as in recent research. Moreover, when the silicon bulk etched array was integrated with active circuitries or interconnection boards [19], the minimal opening for implantation increased. Polymer-constructed arrays utilized various polymer materials to support the 3-D structure [20], but they suffered from process incompatibility with CMOS circuitry. Creating 3-D arrays by the assembly of 2-D parts is now the most popular method to construct a 3-D structure [2,21–25]. The 2-D parts usually include 2-D arrays, vertical spacers and supporting platform. The supporting platform acts as a substrate, and the vertical spacers are erected on the supporting platform by tethers, joints and snap fasteners. The spacers fixed the 2-D arrays vertically on the supporting platform, and made the probe shafts pass through the holes of the supporting platform. The full 3-D structure is therefore like a PC motherboard. Additionally, active circuitry for signal processing can be designed and fabricated in the back-end of the 2-D arrays to achieve system integration. A unique handling method was developed in [24] for a dual-side, ultra-thin silicon substrate process to fabricate thin probe shafts without using doping etching stop technique. Moreover, stacked probes and PCBs by anisotropic conductive film create the connection for the dual-side wire routing and 3D structure. Therefore, each side of probe can be wired out separately. An alternative solution provided in [25] integrated the silicon probe with flexible ribbon cables by using thermosonic bonded gold bump. Also, a platform with bays and gold clips is designed to connect with probes, which results in an impressive 3D device. The comparison of three-dimensional microprobe arrays with some major design parameters is shown in Table 1. However, the studies mentioned above neglect the importance of smaller opening for surgery implantation. Smaller opening of skull can reduce the implantation damage to the subject, prevent the rise of brain pressure, and decrease the infection probability of the wound.

Although previous work creating 3-D arrays by assembly of 2-D arrays successfully achieves high electrode density by packaging active probes onto the supporting platform with some micromechanical packaging technique, some problems still exist. First, previous approaches that use 2-D silicon probes to form full 3-D arrays required complex schemes for assembling submillimeter parts [22]. The main problem of such techniques is that the parts (spacers and supporting platform) were all assembled in orthogonal planes. Thus, perpendicular connectors for interconnections between orthogonal planes were required for signal transmission. Ultrasonic bonding [22] and vertical snap fasteners [2] have been proposed for perpendicular transfer pads, but they suffered from complex assembly steps and precise alignment equipment for 3-D assembly. For example, precise alignment was required to make probe shafts pass through holes of the supporting platform and steady the probe onto the vertical spacers without damage during the assembly process. Second, the probe arrays were fixed only by the perpendicular bonding pads and the tenons. Low structure strength can cause stability problem in implantation. Third, the rooms between the spacers and the 2-D probes were wasted. The volume of a 3-D structure increases rapidly when increasing the number of 2-D probes.

To improve the problems described above, this work reports a new stacking method for fabricating 3-D neural probe arrays. In this study, the 3-D orthogonal interconnection was replaced with 2-D wire bonding by the present stacking method, and the perpendicular bonding and snap fasteners which were used in previous work were no longer needed. Compared to previous work, this new stacking method can also provide reliable structure strength. ASIC chips can be substituted for spacers to increase the system integration and volume usage efficiency as well. Additionally, an anti-overflow design based on the capillary principle was exploited to avoid gel overflow onto proximate bonding pad during 3-D array assembly.

## 2. Design and Method

**Figure 1.**
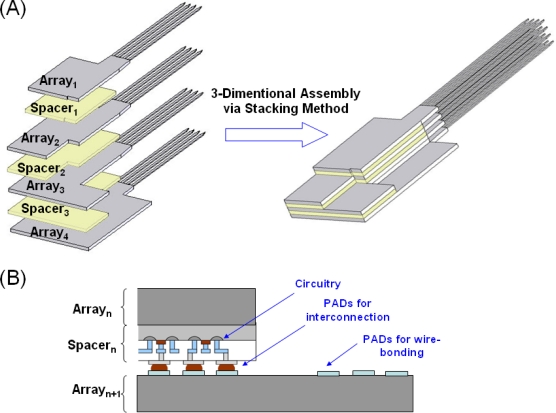
(A) The schematic of stacking a 4 × 4 3-D microprobe array. (B) Spacers can be replaced by silicon substrates with signal processing circuitry for lower fabrication cost, customized design request and increases the volume usage efficiency.

A new stacking method to produce three-dimensional neural probe arrays is presented in this work. This method creates 3-D probe arrays by assembling 2-D arrays and spacers layer by layer, as shown in Figure 1(A). For a 4 × 4 3-D array, four 2-D arrays (gray color) with four probes in each array and three spacers (yellow color) were required. Compared to exising three-dimensional neural probe designs, the present stacking method improved the inconvenient assembly steps which include orthogonal assembly and perpendicular connection techniques. In the stacking method, the shapes of each 2-D arrays were carefully designed so they can be wire-bonded individually with different height levels. Spacers with an anti-overflow mechanism were also proposed in this paper. The present anti-overflow mechanism can also be realized on 2-D arrays if active circuit chips are used as spacers. Also, the thickness of the spacer determined the spacing between two 2-D arrays. Each planar 2-D array, electrode sites, interconnect routing and bonding pads were located in the same plane. The bonding pads were arranged on the different sides of four 2-D probe arrays for wire bonding. Therefore, each 2-D array can be wire-bonded individually and the 3-D perpendicular bonding pads used in previous work are no longer needed.

By replacing spacers with active signal processing circuitry chips, the function of the 3-D neural probe array can be enhanced. For example, as shown in Figure 1(B), active circuit chips can be employed as spacers for signal processing proposes. In Figure 1(B), Spacer_n_ with active circuitry is bonded onto Array_n+1_ by the flip-chip technique, and then Array_n_ is stacked onto Spacer_n_ by adhesion gel. Thus, the 3-D neural probe array can be integrated with circuits by the present stacking method. Additionally, in IC manufacture, larger area means higher cost. When the active circuitry is fabricated with probe shafts in the back-end of the 2-D array, larger wafer areas are required. In the stacking design, spacers with circuitry and 2-D arrays were fabricated individually. Therefore, the stacking method can reduce the cost for circuitry integration and increase the design flexibility when modification of probes/circuitries is required for different applications. Besides, comparing with previous work, the volume usage efficiency was increased because there were no waste rooms between arrays and spacers. In short, the advantages of using active circuit chips as spacers include reducing the cost of circuitry integration, increasing the flexibility of the design and increasing the volume usage efficiency.

## 3. Fabrication of 2-D Probe Arrays and Spacers with Anti-Overflow Mechanism

The fabrication steps of the 2-D array are briefly described as follows: (1) 250 μm-thick silicon wafer was used, and 3 μm-thick polyimide (PI) was spin-coated on the front side of the wafer for electrical isolation. (2) 1 μm-thick Cr/Au layer was electroformed and patterned for wire interconnects on the probe shaft. (3) 3 μm-thick layer polyimide (PI) was spin-coated for the interconnect encapsulation. (4) Electrode sites and wire-bonding pads were defined by DRIE. (5) 3 μm-thick Au was electroformed as electrode site and bonding pad material. Notably, the Au layer was somewhat over-electroformed to ensure that the electrode was in contact with the neural tissue while implantation. (6) The final shape of 2-D probe array was defined and released by DRIE.

The spacers were simply defined on the 250 μm-thick silicon wafer by DRIE. For different application requirements, the thickness of the 2-D arrays and spacers could be modified by using thinner wafer. However, the yield minimal wafer thickness is currently not less than 100 μm due to the accessible process limitation.

When the stacking method is used to construct the 3-D neural probe arrays, the overflow adhesion gel or glue between the stacking layers may cover the proximate bonding pads and make them ineffective. Using less gel may reduce the overflow problem, but reduce the adherent strength. To solve the overflow problem of the gel, an anti-flow mechanism design was applied in the stacking method.

The anti-overflow mechanism was accomplished by creating a through-silicon-via around the edges of the spacers. It uses capillary action force to prevent the gel from overflowing to the bonding pads. The mechanism functions in the following condition: when the stacking process starts, the combined parts compress the adhesion gel and force it to flow around. The flowing glue will fill the via by capillary action as it passes the via. Therefore, there is no redundant glue covering the proximate bonding pads.

The radius of the via was one of the major design parameter in preventing overflow. The formula is given by the well-known capillary action principle [29] with definition of the liquid-air surface tension, contact angle, density of the liquid, acceleration due to gravity, the height of the liquid column and the radius of the via. In this case, the maximal height of the liquid column is the thickness of the spacer (250 μm), and the liquid-air surface tension is 0.033 N/m [30], contact angle is 70° [31], density is 2,000 kg/m^3^ and gravity acceleration is 9.8 m/s^2^. The capillary action principle gives the radius of the via a theoretical result of 4,600 μm, which was even larger than the size of the spacer. In fact, there is some limitations should be put into consideration. For instance, the limited volume of glue, the viscosity of glue, the friction force between glue-substrate interface and the capillary force in the narrow gap between two parts will make the liquid column never reach the expected height. The final via radius was experimentally set as 250 μm to enhance the filing of the via with glue.

**Figure 2.**
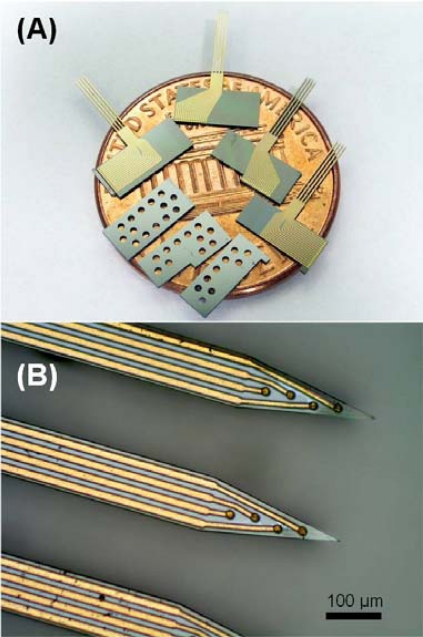
Microphotographs of fabricated 2-D arrays. (A) Fabricated parts on a one cent coin. (B) Probe tip and electrode sites. The tapered tip angle is about 23°.

Figure 2 shows the assembly parts successfully manufactured by the fabrication steps as described above. Figure 2(A) presents the fabricated parts for 3-D assembly on a one cent coin. Figure 2(B) displays the probe tip and electrode sites. Each 2-D probe array consisted of four shafts of 6 mm length, 100 μm width and the space between shafts was 110 μm. Four electrode sites were lined up on the tip along the shaft, separated by 50 μm, and the first electrode is 70 μm from the shaft tip. The circular electrode had a diameter of 20 μm and the square bonding pad had sides of length 80 μm. The tapered tip angle was around 23°. The circular via on spacers for the anti-overflow mechanism can be observed in the picture.

## 4. 3-D Probe Array Assembly

After the assembly parts were successfully fabricated, a flip-chip bonder (FINETECH Inc., USA) and thermosetting polymer (EA2151, LIONTONG Inc., TAIWAN) were used to complete the 3-D neural probe array assembly process. Convenient flip-chip technology was employed to accomplish alignment, pressurization and heating process, while the thermosetting polymer (glue) provided adhesive layer between two stacked components (array and spacer). The thermosetting glue was solidified at 185 °C in 180 s with an adhesive strength of 150–180 kg/cm^3^. The detailed steps to manufacture a 4 × 4 3-D neural probe array (as shown in Figure 1) using the tools as described above were as follows:

1. Array_4_ was fixed on the vacuum holder of the flip-chip bonder.
2. Second, adhesion gel was deposited onto the Array_4_ by a micro injector (MICRO FAB TECH. Inc., USA).
3. Spacer_3_ was picked by the flip-chip bonder head and aligned with the 2-D array by an optical microscope.
4. Then, Array_4_ & Spacer_3_ were bonded together in a pressurizing and heating condition provided by the flip-chip head.
5. After the first bonding process finished, the flip-chip head was released from the Spacer_3_, and then adhesion gel was deposited onto Spacer_3_ by the micro injector.
6. Next, Array_3_ was picked, aligned and bonded to the Spacer_3_ again.
7. Repeating the bonding process 1 to 6, we accomplished the 4 × 4 3-D neural probe array.

The maximal placement accuracy of the flip-chip was 0.5 μm in a single bonding step. Thus, the total miss-alignment error can be neglected. Moreover, the average assembly time for a 4 × 4 3-D microprobe array by manual alignment was approximately 35 minutes (including heat curing time). In the present study, we applied about 0.26 μL of gel between spacers and arrays. The appropriate amount of the adhesion get combined the stacking well without spilling to the proximate pads.

**Figure 3.**
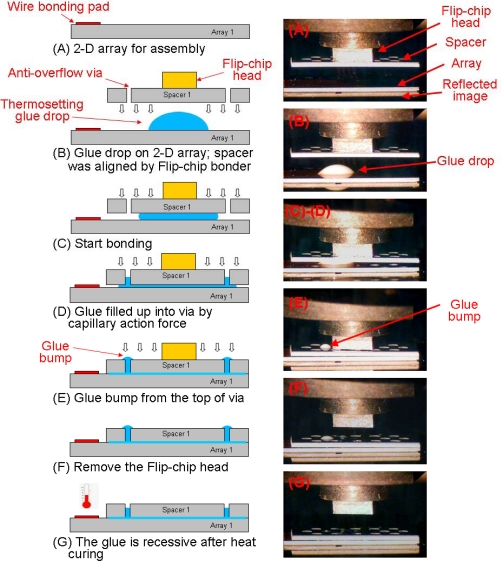
The proposed assembly anti-flow mechanism process and related practical photographs.

Figures 3(A)–(G) illustrate how the anti-overflow mechanism functions in the practical assembly process. The details are displayed as follows: (A) the fabricated 2-D array (Array_n_) was fixed on the flip-chip holder (not shown). (B) A drop of thermosetting polymer was deposited onto the 2-D probe array and the spacer (Spacer_n_) was picked by the flip-chip bonder head and aligned. (C) Start bonding—the aligned spacer was moved downward and controlled by the flip-chip bonder head. After the spacer came into contact with the glue drop, the drop spread in random directions because it was squeezed by the spacer. (D) The spacer was moved continuously downward, and the glue filled the via by capillary force when it flowed past the via. (E) The spacer came into contact with the 2-D array. The gel bump occurred on the top of the via because the pressure from bonding. (F) The flip-chip bonding head was removed. (G) The assembly process was completed following thermal solidification of the thermosetting glue. The gel bump over the via rapidly receded after heat curing.

**Figure 4.**
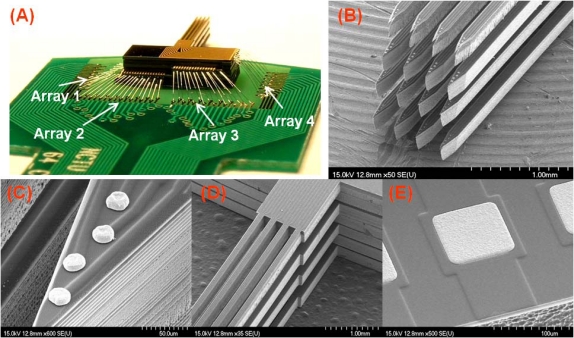
The photographs of successfully assembled 3-D microprobe array. (A) The wire-bonded result of 3-D microprobe array. Four different bonding levels were marked. (B) Close view of 4 × 4 shafts. (C) The electrodes sites located at the shaft tip. (D) The cantilever shaft structure. (E) Pad for wire bonding.

After applying the steps described in Figure 3, we successfully assembled 3-D microprobe arrays, as shown in Figure 4. Figure 4(A) displays stacked 3-D microprobe array was mounted and wire-boned onto a pre-designed PCB (∼600 μm in thickness). Figure 4(B) shows a close view of 4 × 4 shafts. Figure 4(C) presents the electrodes sites at the shaft tip. Figure 4(D) illustrates the cantilever shaft structure. Figure 4(E) shows the pad for wire bonding (without wire-bonding). The electrode sites on the probe shafts were over-electroformed to ensure that the electrode can come into contact with neural tissue during implantation. Figure 4(A) also shows that the 3-D signal transmission was achieved by 2-D wire-bonding with four level of bonding pads (Array_1_, Array_2_, Array_3_ and Array_4_) in a 4 × 4 3-D arrays.

## 5. Characterization of 3-D Probe Array

Electrode impedance spectroscopy (EIS) was used to evaluate the fabricated stacked 3-D probe arrays. The impedance characterization of 3-D neural probe array in the electrode-electrolyte interface is of utmost importance in impedance-based biosensing and neuroprotheses [32]. When the electrode sites come into contact with tissue, an electrode-tissue interface impedance was established. The electrode-tissue interface impedance and the amplifier input impedance act as a voltage divider when a neural signal passes through the electrode into the front-end amplifier. Hence, high electrode-tissue interface impedance will cause signal attenuation and induce considerable thermal noise in weak raw signal recording.

**Figure 5.**
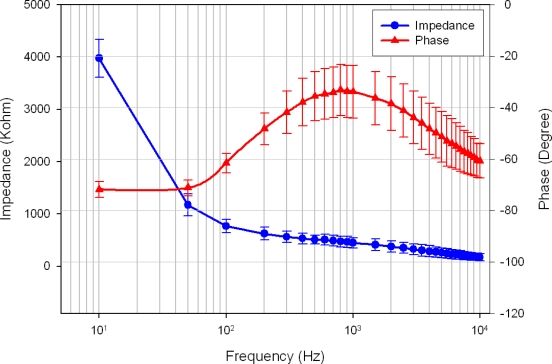
The electrode impedance spectroscopy of fabricated microprobe array in physiologic saline solution. Means and standard deviations are given (n = 16).

The final assembled array was characterized in physiologic saline solution (0.9% NaCl) at room temperature using a multi-frequency LCR meter (Wayne Kerr LCR meter 4235). Figure 5 presents the measured impedance of the electrodes (n = 16) on the microprobe. The *in-vitro* impedance was 463 ± 107 kΩ and the phase was −33 degree at 1 kHz.

## 6. Neural Recording

**Figure 6.**
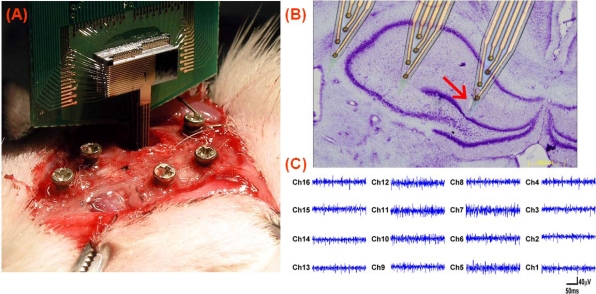
(A) Photograph of a stacked 3-D microprobe array inserted into the brain of an anesthetized rat. (B) The *in situ* location of microprobe array was shown in the photomicrograph Nissl-stained coronal section. (C) The 16-channel neural activities simultaneously recorded from CA1 in of hippocampus.

To demonstrate the practical function, the fabricated 3-D neural probe array was implanted into an anesthetized rat. Figure 6(A) shows the photograph of a stacked 3-D microprobe array that was inserted into the brain of an anesthetized rat by a manual 3-axis moving stage (not shown in the figure). The screw on the skull was adopted as a reference for measurement. The opening in the skull was about 2 mm by 3 mm. Figure 6(B) shows the photomicrograph of the implantation section. The figure was modified by superimposing a lesion marker, an implantation track, and overlaying a scaled image of the microprobe array. One lesion marker arrowhead (red) was used to identify the location of the outermost recording site, in relation to the field CA1 of the hippocampus. Figure 6(C) presents neural signals from the 16-channel microprobe array, acquired with a Multi-Channel Acquisition Processor (MAP, Plexon Inc., USA). During recordings, electrical signals were passed from the headstage to an amplifier through a band-passed filtered (spike preamp filter: 450–5 kHz, gain: 15,000–20,000) and sampled at 40 kHz per channel.

## 7. Discussion

The minimal opening is the area that must be resected, including skull and dura, to fully place an implantable device onto the brain. A smaller skull opening can reduce the implantation damage such as the rise of intracranial pressure, and the probability of wound infection. In previous work, the opening area was never less than the supporting platform [22,23] to make sure all the probes were completely inserted into tissue. Therefore, the minimal surgical opening area was defined by the supporting platform in this case. Additionally, the supporting platform area was significantly increased when ASIC chips were mounted onto the platform for system integration [2]. In the proposed stacking method, the system integration will not increase the opening area because it can be accomplished by replacing spacers with ASIC chips. The opening area of present 3-D probe array depends only on the probe array dimension. The minimum opening area of the stacked 3-D probe array is less than 1.75 mm × 1 mm, which can be readily shrunk by using thinner and narrow shafts.

Previous work may also induce additional tissue damage in the bottom of the platform as well. The interlocking structures, including tethers and joints, can cause the protrusion and damage to the tissue underneath [2]. For the stacked 3-D array, only probe array will be in contact with the target tissue.

The strength of the assembled structure is also an important issue in implantable device. Compared with the proposed 3D array, structures with tethers and joints used in previous work may not provide reliable strength to fix the probes on the platform during implantation [2,22]. The thermosetting polymer in the stacked 3-D array provided an adhesive strength of 150–180 kg/cm^3^ after curing. Thus, sufficiently structural strength was guaranteed in the present design.

In summary, compared with previous 3-D array studies, the advantages of using the stacking method for constructing 3-D arrays include easier assembly processes, stronger structure strength, smaller opening area and less damage to the tissue surrounding the implanting region. ASIC chips can be substituted for spacers to achieve system integration without increasing device size as well. The stacking method can therefore increase the design flexibility and enhance the volume usage efficiency.

## 8. Conclusions

In this work, a new assembly method was applied to design a 3-D neural probe array. The fabrication and test results were also presented. The proposed stacking method can replace the vertical interconnections used in previous work with 2-D wire bonding. In this way, the supporting platform with slots and vertical spacers was no longer needed. To avoid the fluid overflow during assembly, an anti-overflow design which made use of capillary action force was applied in the stacking method as well. Furthermore, ASIC chips can also be substituted for the spacers in the stacked arrays to achieve system integration, design flexibility and volume usage efficiency. The time for manually assembling a 4 × 4 3-D microprobe array was approximately 35 minutes. Compared with previous 3-D array studies, the advantages of using the stacking method for constructing 3-D arrays include easier assembly processes, stronger structure strength, smaller opening area and less damage to the tissue surrounding the implanting region. Practical *in-vivo* neural spike recordings also demonstrated the functionality of the proposed neural probe array.

## Figures and Tables

**Table 1.** Comparison of three-dimensional microprobe arrays with some major design parameters.

**Reference**	[18,19]	[26–28]	[20]	[2,21–23]	[25]	[24]
**Substrate**	Si	Epoxy, Polyimide	Polyimide/Nickel	Si	Si	Si
**Dimension**	3D	3D	3D	3D	3D	3D
**3D method**	Bulk silicon etching, out of plane	Molded tooling/hand made	Bulk silicon etching, out of plane	Slots, platform, vertical spacer	Stacking with PCB	Platform with bays
**Electrode material**	Al	Tungsten/SiC	Al/Ti	Ir	Au	Au
**Electronics compatibility**	Yes (by Stacking & Wire-bonding)	No	No	Yes (Embedded in back-end/platform)	--	--
**Number of electrode per shaft/Number of shaft/Number of total sites**	1/100/100 or 1/16/16	1/33/33 or 1/16/16	3/6/18	4/16/64 or 4/128/512 or 8/32/256	8/3/24	5/16/80
**Shaft length (mm)**	1.5	3–5	1.2	1.2, 2.5, 3.3	5	2
**Shaft width (um)**	90	50, 90, 120	160	40, 50, 144	90	--
**Shaft thickness (um)**	90	50, 90, 120	26	12–100	50	100
**Shaft spacing (um)**	400	250, 400, 450	450	200, 256	90	--
**Electrode size (um^2^)**	--	--	400	81, 100, 1000	100	--
**Electrode spacing (um)**	--	--	200	24, 400	30	--
**Back-end size (mm^2^)**	6.35 × 6.35	--	--	5.7 × 4	--	∼5 × 5
**Minimal opening required for implantation (mm^2^)**	>6.35 × 6.35–1.56 × 1.56	>3.3 × 1.05, 1.56 × 1.56	>1.9 × 2	>2.5 × 4.8, 5.7 × 4	--	>5 × 5
**Structure strength**	High	Medium to high	Low	Low	Medium	Medium
**Remarks**	Dicing saw defined probe array	3D structure by epoxy supporting	Magnetic batch assembly	Ultra-sonic for wiring and Low profile structure	Anisotropic conductive film is used	Thermosonic bonding with ribbon cable
