# Correction: Chang, C.W., *et al*. Development of a Three Dimensional Neural Sensing Device by a Stacking Method. *Sensors* 2010, *10*, 4238–4252

**DOI:** 10.3390/s100504716

**Published:** 2010-05-07

**Authors:** Chih-Wei Chang, Jin-Chern Chiou

**Affiliations:** 1 Department of Electrical Engineering, National Chiao Tung University, No. 1001, University Road, Hsinchu City, Taiwan; E-Mail: cwchang.ece94g@nctu.edu.tw; 2 School of Medicine, China Medical University, No.91, Hsueh-Shih Road, Taichung, Taiwan

The authors would like to correct the affiliations and acknowledgement of this paper [1] as follows:

^1^ Department of Electrical Engineering, National Chiao Tung University, No. 1001, University Road, Hsinchu City, Taiwan; E-Mail: cwchang.ece94g@nctu.edu.tw

^2^ School of Medicine, China Medical University, No.91, Hsueh-Shih Road, Taichung, Taiwan
